# Effects of Medicare Advantage Growth in Nursing Homes on Care Quality and Resident Outcomes: Using Shift-Share IV

**DOI:** 10.1093/geroni/igaf122.3428

**Published:** 2025-12-31

**Authors:** Hyunkyung Yun, David Meyers, Vincent Mor, Cyrus Kosar, Peter Hull, Hye-Young Jung, Momotazur Rahman

**Affiliations:** Brown University, Providence, Rhode Island, United States; Brown University, Providence, Rhode Island, United States; Brown University School of Public Health, Providence, Rhode Island, United States; Brown University, Providence, Rhode Island, United States; Brown University, Providence, Rhode Island, United States; Joan and Sanford I. Weill College of Medicine, Cornell University, New York, New York, United States; Brown University, Providence, Rhode Island, United States

## Abstract

Medicare Advantage (MA) now covers 54% of all Medicare beneficiaries. MA growth has expanded to nursing home (NH) setting, covering 37% of short-stay patients and 32% of long-stay residents in 2021. The impact of MA growth in NHs remains understudied. Using a shift-share instrumental variable (SSIV), we estimated effects of MA growth in NHs on care quality. We identified MA enrollment of Medicare-enrolled NH residents using the Medicare enrollment file (2015-2019). NH quality measures included CMS five-star overall and staffing ratings and health deficiencies. Population-specific outcomes, derived from claims, included proportion of short-stay residents with 30-day rehospitalization and death, and the quarterly rate of hospitalization and death among long-stay residents. The main explanatory variable was high MA share in NHs (top quartile NH MA share annually). We used a SSIV, leveraging an exogenous shift—the annual national MA contract growth—linked to baseline NH contract share. We estimated a two-stage least squares (2SLS) regression with NH and year fixed effects, adjusting for NH and county characteristics and clustering standard errors for NHs. Among 18,711 NHs (83,215 observations over five years), 31% had high MA share. The first-stage F-statistic was 528.7 (Coef. 0.12, P < 0.001), confirming IV strength. In 2SLS results, high MA share in NHs was associated with a 0.6 increase in staffing ratings (95% CI 0.4-0.9; P < 0.001), an 18.8% relative increase, but not with overall star-rating, deficiencies, or population-specific outcomes. Although these results suggest MA growth has improved staffing levels at NH, the mechanisms underlying these findings warrant further investigation.

